# Supplementary material for: Short-Chain Fatty Acid Reference Ranges in Pregnant Women from a Mediterranean Region of Northern Spain: ECLIPSES Study
Source: Nutrients. 2022 Sep 15;14(18):3798. doi: 10.3390/nu14183798 (PMC9503449; doi:10.3390/nu14183798)
Supplement: Supplementary file 1 [file nutrients-14-03798-s001.zip › nutrients-1858481-supplementary.pdf]

**Supplementary Table S1**. Multivariate- adjusted linear regression of potential maternal factors related to maternal serum short chain fatty acids composition in the first trimester of pregnancy.

| | Short chain fatty acids | | | | | | | | | | | |
|---|---|---|---|---|---|---|---|---|---|---|---|---|
| | Acetic acid (C2:0) | | | Propionic acid (C3:0) | | | Isobutyric acid (C4:0) | | | Butyric acid (C4:0) | | |
| Maternal factors | β | SE | *p* | β | SE | *p* | β | SE | *p* | β | SE | *p* |
| Age (years) | -0.52 | 0.29 | 0.074 | -0.01 | 0.01 | 0.550 | -0.00 | 0.00 | 0.438 | -0.00 | 0.00 | 0.717 |
| Gestational weight gain (kg) | -0.27 | 0.36 | 0.444 | 0.00 | 0.02 | 0.943 | -0.00 | 0.00 | 0.928 | -0.01 | 0.01 | 0.227 |
| BMI (25-29.9 (ref.) *vs.* <25 kg/m$^2$) | -2.75 | 3.05 | 0.367 | -0.03 | 0.13 | 0.800 | 0.02 | 0.03 | 0.593 | -0.00 | 0.05 | 0.973 |
| BMI (25-29.9 (ref.) *vs.* ≥30 kg/m$^2$) | 0.02 | 4.23 | 0.997 | 0.09 | 0.18 | 0.635 | -0.00 | 0.04 | 0.979 | -0.06 | 0.06 | 0.326 |
| Educational level | 0.21 | 1.98 | 0.917 | -0.01 | 0.08 | 0.945 | 0.01 | 0.02 | 0.725 | -0.04 | 0.03 | 0.215 |
| Social class | 0.01 | 2.76 | 0.997 | 0.02 | 0.12 | 0.837 | 0.00 | 0.03 | 0.953 | -0.02 | 0.04 | 0.702 |
| Parity (primiparous (ref.) *vs.* multiparous) | -1.38 | 2.75 | 0.617 | 0.07 | 0.12 | 0.529 | 0.00 | 0.03 | 0.928 | -0.06 | 0.04 | 0.134 |
| Smoking during pregnancy (no (ref.) *vs.* yes) | -4.57 | 3.49 | 0.192 | -0.26 | 0.15 | 0.075 | -0.01 | 0.03 | 0.778 | -0.01 | 0.05 | 0.886 |
| PA, METs/week (moderate (600–2999) (ref.) *vs.* low (<600)) | -2.21 | 2.84 | 0.438 | 0.07 | 0.12 | 0.563 | 0.02 | 0.03 | 0.437 | -0.06 | 0.04 | 0.201 |
| PA, METs/week (moderate (600–2999) (ref.) *vs.* high (≥3000)) | -4.32 | 6.16 | 0.484 | -0.32 | 0.26 | 0.225 | -0.00 | 0.06 | 0.937 | -0.14 | 0.09 | 0.132 |
| SQDI (score) at first trimester | -1.15 | 0.67 | 0.088 | -0.02 | 0.03 | 0.399 | -0.01 | 0.01 | 0.118 | -0.03 | 0.01 | **0.006** |
| Alcohol consumption (no (ref.) *vs.* yes) | 1.94 | 22.21 | 0.930 | 0.10 | 0.94 | 0.919 | 0.09 | 0.22 | 0.660 | -0.08 | 0.34 | 0.812 |
| Energy intake (kcal/d) at first trimester | -0.01 | 0.01 | 0.574 | 0.00 | 0.00 | 0.506 | -0.00 | 0.00 | 0.816 | -0.00 | 0.00 | 0.072 |
| Total protein intake (g/d) at first trimester | 0.08 | 0.17 | 0.647 | -0.00 | 0.01 | 0.761 | -0.00 | 0.00 | 0.970 | 0.00 | 0.00 | 0.329 |
| Fibre intake (g/d) at first trimester | 0.54 | 0.80 | 0.499 | -0.02 | 0.03 | 0.656 | 0.00 | 0.01 | 0.599 | 0.03 | 0.01 | **0.022** |
| | $R^2$ = 0.045, $F_{15,270}$= 0.85; p= 0.622 | | | $R^2$ = 0.038, $F_{15,270}$= 0.73; p= 0.755 | | | $R^2$ = 0.020, $F_{15,268}$= 0.98; p= 0.985 | | | $R^2$ = 0.070, $F_{15,268}$= 1.35; p= 0.169 | | |

Multivariate linear regression models were used to calculate β coefficient (β) and standard error (SE). The models were mutually adjusted for all characteristics displayed in this table. The significance of numbers in bold is p-value<0.05. Abbreviations: BMI, body mass index; PA, physical activity; METs, metabolic equivalents.

**Supplementary Table S2**. Multivariate- adjusted linear regression of potential maternal factors related to maternal serum short chain fatty acids composition in the third trimester of pregnancy.

| | **Short chain fatty acids** | | | | | | | | | | | |
|---|---|---|---|---|---|---|---|---|---|---|---|---|
| | **Acetic acid (C2:0)** | | | **Propionic acid (C3:0)** | | | **Isobutyric acid (C4:0)** | | | **Butyric acid (C4:0)** | | |
| **Maternal factors** | **β** | **SE** | ***p*** | **β** | **SE** | ***p*** | **β** | **SE** | ***p*** | **β** | **SE** | ***p*** |
| Age (years) | 0.24 | 0.26 | 0.370 | -0.00 | 0.02 | 0.907 | 0.00 | 0.00 | 0.245 | 0.00 | 0.01 | 0.501 |
| Gestational weight gain (kg) | -0.48 | 0.34 | 0.153 | -0.03 | 0.02 | 0.147 | -0.00 | 0.00 | 0.419 | 0.01 | 0.01 | 0.307 |
| BMI (25-29.9 (ref.) *vs.* <25 kg/m$^2$) | -0.90 | 2.79 | 0.746 | -0.23 | 0.18 | 0.200 | -0.02 | 0.04 | 0.539 | 0.00 | 0.07 | 0.992 |
| BMI (25-29.9 (ref.) *vs.* ≥30 kg/m$^2$) | 0.52 | 4.02 | 0.897 | 0.28 | 0.26 | 0.289 | -0.03 | 0.05 | 0.640 | 0.08 | 0.10 | 0.425 |
| Educational level | -1.00 | 1.76 | 0.569 | 0.04 | 0.11 | 0.719 | -0.03 | 0.02 | 0.210 | -0.03 | 0.04 | 0.505 |
| Social class | 1.16 | 2.51 | 0.643 | -0.08 | 0.16 | 0.606 | 0.05 | 0.03 | 0.177 | -0.06 | 0.06 | 0.345 |
| Parity (primiparous (ref.) *vs.* multiparous) | 0.68 | 2.46 | 0.782 | 0.18 | 0.16 | 0.248 | -0.02 | 0.03 | 0.490 | -0.05 | 0.06 | 0.410 |
| Smoking during pregnancy (no (ref.) *vs.* yes) | -1.06 | 3.17 | 0.739 | -0.18 | 0.20 | 0.377 | -0.08 | 0.04 | 0.054 | -0.16 | 0.08 | **0.046** |
| PA, METs/week (moderate (600–2999) (ref.) *vs.* low (<600)) | 3.48 | 2.56 | 0.175 | 0.37 | 0.16 | **0.026** | 0.08 | 0.03 | **0.020** | -0.05 | 0.07 | 0.434 |
| PA, METs/week (moderate (600–2999) (ref.) *vs.* high (≥3000)) | 2.14 | 5.00 | 0.668 | 0.14 | 0.32 | 0.672 | -0.02 | 0.07 | 0.747 | -0.07 | 0.13 | 0.582 |
| SQDI (score) at third trimester | 0.21 | 0.60 | 0.727 | 0.02 | 0.04 | 0.576 | -0.02 | 0.01 | 0.052 | -0.00 | 0.02 | 0.751 |
| Alcohol consumption (no (ref.) *vs.* yes) | -14.47 | 18.32 | 0.431 | -1.15 | 1.17 | 0.326 | -0.16 | 0.25 | 0.528 | -0.02 | 0.47 | 0.968 |
| Energy intake (kcal/d) at third trimester | 0.01 | 0.01 | 0.297 | 0.00 | 0.00 | 0.737 | -0.00 | 0.00 | 0.966 | -0.00 | 0.00 | 0.941 |
| Total protein intake (g/d) at third trimester | -0.21 | 0.16 | 0.200 | -0.01 | 0.01 | 0.363 | -0.00 | 0.00 | 0.297 | 0.00 | 0.00 | 0.904 |
| Fibre intake (g/d) at third trimester | -0.26 | 0.66 | 0.690 | 0.00 | 0.04 | 0.932 | 0.01 | 0.01 | 0.431 | -0.00 | 0.02 | 0.874 |
| | $R^2$ = 0.045, $F_{15,219}$= 0.51; p= 0.936 | | | $R^2$ = 0.084, $F_{15,217}$= 1.31; p= 0.187 | | | $R^2$ = 0.109, $F_{15,218}$= 1.78; p= **0.038** | | | $R^2$ = 0.039, $F_{15,216}$= 0.59; p= 0.883 | | |

Multivariate linear regression models were used to calculate β coefficient (β) and standard error (SE). The models were mutually adjusted for all characteristics displayed in this table. The significance of numbers in bold is p-value<0.05. Abbreviations: BMI, body mass index; PA, physical activity; METs, metabolic equivalents.
